# Toxicological Effects of Berberine and Sanguinarine

**DOI:** 10.3389/fmolb.2018.00021

**Published:** 2018-03-19

**Authors:** Nitika Singh, Bechan Sharma

**Affiliations:** Department of Biochemistry, Faculty of Science, University of Allahabad, Allahabad, India

**Keywords:** berberine, sanguinarine, alkaloids, toxicity, pharmacological properties

## Abstract

Berberine and Sanguinarine alkaloids belong to a group of naturally occurring chemical compounds that mostly contain basic nitrogen atoms. This group also includes some related compounds with neutral or weakly acidic properties. Alkaloids are produced by a large number of organisms including bacteria, fungi, plants, and animals. Berberine and Sanguinarine both are isoquinoline derivatives and belong to protoberberine and benzophenanthridines, respectively. Tyrosine or phenylalanine is common precursor for the biosynthesis of both. Sanguinarine [13-methyl (1,3) benzodioxolo(5,6-c)-1,3-dioxolo (4,5) phenanthridinium] is a toxin that kills animal cells through its action on the Na^+^-K^+^-ATPase transmembrane protein. Berberine, on the other hand, has been reported to cause cytotoxicity and adversely influence the synthesis of DNA. Several workers have reported varied pharmacological properties of these alkaloids as they exhibit antibacterial, antiasthma, anticancer, anti-inflammatory, and antidiabetic activities. This review article illustrates the toxicological effects of berberine and sanguinarine as well as mechanistic part of berberine and sanguinarine mediated toxicity in different living systems. This manuscript has included the lethal doses (LD_50_) of berberine and sanguinarine in different animals via different routs of exposure. Also, the effects of these alkaloids on the activities of some key enzymes, cell lines and organ development etc. have been summarized.

## Introduction

**Figure 1:**
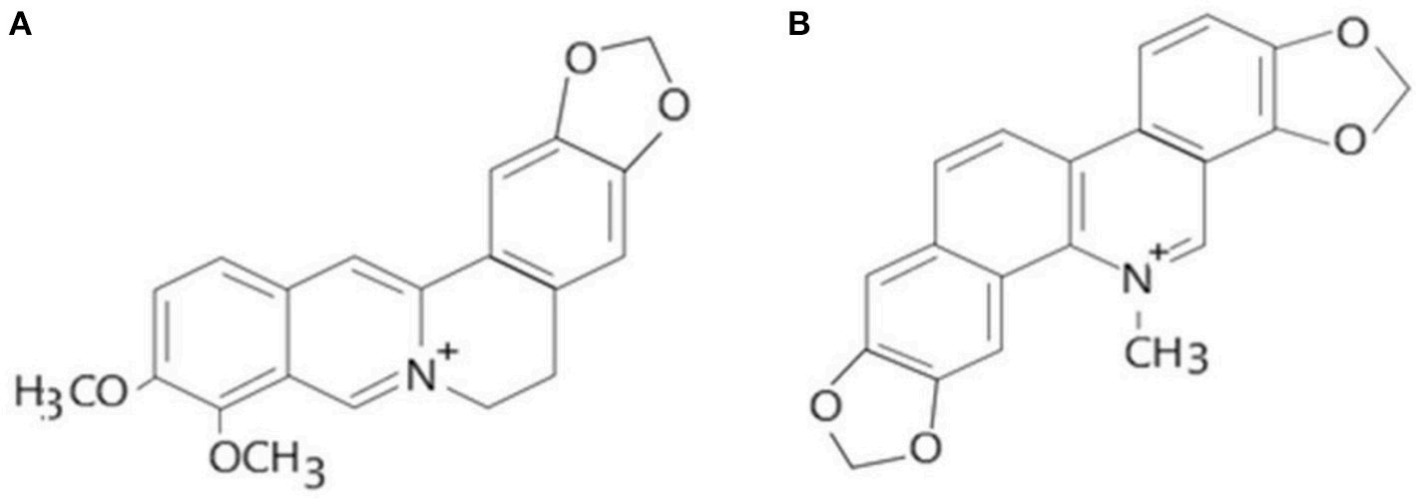
Molecular structures of berberine **(A)** and sanguinarine **(B)** (Source: Hao et al., 2014).

Alkaloids are a group of naturally occurring chemical compounds that mostly contain basic nitrogen atoms (Figure 1). Sometimes alkaloids also include some related chemical compounds with weakly acidic and neutral properties. A large variety of organisms produces (bacteria, fungi, plants, and animals) alkaloids that can be purified by acid-base extraction from crude extracts of these organisms. A number of pharmacological activities such as antimalarial (e.g., quinine), antiasthma (e.g., ephedrine), anticancer (e.g., homoharringtonine), cholinomimetic (e.g., galantamine), vasodilatory (e.g., vincamine), antiarrhythmic (e.g., quinidine), analgesic (e.g., morphine), antibacterial (e.g., chelerythrine), and antihyperglycemic activities (vincristine and vinblastine) of alkaloids has been reported. Berberine and sanguinarine both are isoquinoline derivatives and belong to protoberberines and benzophenanthridienes, respectively. Some chelerythrine isolated from *Argemone mexicana* alkaloids such as *N*-demethyloxysanguinarine, pancorine, (+)-argenaxine, (+)-higenamine, (+)-reticuline, angoline, and chelerythrine isolated from *A. mexicana* and have been reported for their cytotoxic activities against human nasopharyngeal carcinoma (HONE-1) and human gastric cancer (NUGC) cell lines (Chang et al., 2003). Berberamine, berberine, palmatine, columbamine, oxyberberine, isocorydine, lambertinea, and magniflorine have been isolated from different species of berries (*Berberies vulgaris, Berberies candidula* etc.). These alkaloids are reported to exert anti-cancer, anti-inflammatory, antioxidant, antidiabetic, antibacterial, analgesic and anti-nociceptive, and hepatoprotective effects. Several pharmacological activities of berberine and sanguinarine are similar. The aromatic amino acid such as tyrosine or phenylalanine is a common precursor for the biosynthesis of both. The molecular formula of berberine and sanguinarine are C_20_H_19_NO5 and C_2_H_15_NO_5_, respectively (Chao et al., 2013; Yatoo et al., 2018).

Berberine, an isoquinoline alkaloid, belongs to the class of protoberberine alkaloids (Ikuta and Itokawa, 1988). The genus Berberis with more than 500 species belongs to Berberidaceae family (Rounsaville and Ranney, 2010). Berberine is also present in plants of Papaveraceae and Ranunculaceae families. Berberies are an evergreen shrub which possesses yellow, spiny, angled or sulcated bark, oblong, obovate, or elliptic leaves, yellow flowers and red, oblong fruits (Ahrendt, 1961). Berberine is crystal bright yellow in color and present in different parts such as roots, stem, bark, rhizome, fruit and leaves (rarely) of several plant species (mostly in barberry), the *Thalictrum rochebrunianum* (meadow rue), the *Chelidonium majus* (celandine), the *Hydrastis canadensis* (goldenseal), and the *Phellodendron amurense* (Amur cork tree), etc. Among these berberine are mainly present in a variety of barberry species and goldenseal species which are native to Asia and America, respectively (Manske and Holmes, 1995). Some workers have reported berberine (5.2–7.7%) as a major active component of *Rhizoma coptidis* (Huang Lian) which is traditional herb of china (Yina et al., 2012). First time in 1988 hypoglycemic effect of berberine has been reported during the treatment of diarrhea in diabetic patients. Since then, berberine as an anti-diabetic agent has been used on large scale and known as folk medicine of China. A number of research workers reported the use of this alkaloid in treatment of various diseases including problems in cardiovascular, endocrine, gastrointestinal, renal and central nervous system (Imanshahidi and Hosseinzadeh, 2008). Recent publications demonstrate the anti-oxidant (Abd El-Wahab et al., 2013), anti-inflammatory (Lin et al., 2013), anti-tumor (Yu et al., 2007), anti-mutagenic (Cernakova et al., 2002), and anti-diabetic (Abd El-Wahab et al., 2013) properties of berberine. The potential antitumor activity of berberine hydrochloride has always been a subject of considerable interest because of the known capability of berberine to bind with nucleic acids. Its ability to bind specifically to oligonucleotides and to stabilize DNA triplexes or G-quadruplexes via telomerase and topoisomerase inhibition accounts for its antiproliferative activity (Tan et al., 2011; Hao et al., 2014). In addition, berberine is reported to induce a significant hormetic dose response, in which the low dose of berberine strongly stimulates the growth of cancer cells, while at high doses it acts as anticancer agents (Bao et al., 2015). Moreover, its extensive occurrence in various plant species and low toxicity suggest that berberine hydrochloride has the potential to become an effective antitumor agent in future.

Sanguinarine [13-methyl (1,3) benzodioxolo (5,6-c)-1,3-dioxolo (4,5) phenanthridinium] derived from the root of *Sanguinaria canadensis* and other poppy-fumaria species of Papaveraceae family, is the most widely used benzophenanthridine alkaloid (Laster and Lobene, 1990). Sanguinarine is a benzophenanthridine structural homolog of chelerythrine (Pi et al., 2008). A positive moiety is present in the aromatic ring of the molecule. In similarity to berberine, sanguinarine also have antimicrobial, antioxidant and anti-inflammatory properties (Firatli et al., 1994). The cytotoxic and cytostatic effects of sanguinarine on a variety of human cancer cells, including human epidermoid carcinoma, erythroleukemia, prostate cancer, pancreatic carcinoma, colon cancer, breast cancer, lung cancer, promyelocytic leukemia, and bone cancer (Weerasinghe et al., 2001a,b; Matkar et al., 2008; Vrba et al., 2009; Park et al., 2010), have been reported. Sanguinarine exhibits the highest cytotoxicity among benzophenanthridine alkaloids (Slaninová et al., 2001; Vogel et al., 2010).

Sanguinarine is a toxin that kills animal cells through its action on the Na^+^-K^+^-ATPase transmembrane protein. The normal physiological functions of Na^+^-K^+^-ATPase are to maintain the resting potential and to regulate cellular volume by pumping sodium out of cells and potassium into the cells, both against their concentration gradients. The pumping of Na^+^-K^+^ is mediated through active transport, uses energy in the form of ATP and also plays significant role in cell physiology. The Na^+^-K^+^-ATPase is known to play key role in regulating mitogen activating protein kinases (MAPK) pathway, reactive oxygen species (ROS) and intracellular calcium by acting as signal transducer. Thus, the sanguinarine mediated toxicity to Na^+^-K^+^-ATPase can result into development of abnormal cellular functions (Pitts and Meyerson, 1981; Horisberger and Geering, 2009).

Epidemic dropsy is a disease that results from ingesting sanguinarine. Benzophenanthridine sanguinarine alkaloids involves cell death signaling pathway and apoptosis induction mechanism in cancer cell lines. Induction of apoptosis by sanguinarine targeted through mitochondrial damage, nuclear factor kappa-light-chain enhancer of activated B cells activation, and cell cycle arrest (Malikova et al., 2006b). Sanguinarine has been reported to inhibit microtubule polymerization and benzophenanthridine cytotoxic activity involves intercalation of double-stranded deoxyribonucleotide (DNA) (Lopus and Panda, 2006; Matkar et al., 2008) and induces fragmentation of DNA. Recent studies stated that the cytotoxicity and DNA damaging effect of sanguinarine is more specific to cancer cells than to normal cells (Ahmad et al., 2000; Matkar et al., 2008). Cell death mechanism of sanguinarine particularly involves cytotoxic and apoptotic effects occurring by changing the apoptotic gene expressions in human SH-SY5Y and Kelly neuroblastoma cell lines (Cecen et al., 2014). Toxicity of sanguinarine (other than inhibition of Na^+^/K^+^ATPase) have been explained in the form of cell membrane damage by lipid peroxidation by free radicals including ROS and r active nitrogen species (RNS). It also indicates DNA polymarase activity inhibition and accumulation of pyruvate due to increased glycogenolysis (Verma et al., 2001). Sanguinarine inhibits the growth of tumor through different molecular pathways. Sanguinarine also inhibits the proliferation and invasiveness of tumor cells i.e., called as the complex phenomena of tumor angiogenesis. In particular, owing to its pro-apoptotic potential, sanguinarine is a good candidate for the development of new anticancer therapeutics either when used alone or in combination with other chemotherapeutic regimens (Gaziano et al., 2016).

Keeping in view the toxicological implications of these two plant-based alkaloids i.e., berberine and sanguinarine, it was considered imperative to update the information on their impacts on the biochemical, cellular and molecular indices in biological systems. The rational for choosing the berberine and sanguinarine (Figure 1) for their toxicological properties are its structural similarity and both are belonging to the isoquinoline group. The structural formulas of berberine and sanguinarine molecules are C20H19NO5 and C20H15NO5, respectively. Usually, isoquinoline alkaloids interact with DNA as intercalators, or they are arranged in a small groove; their external binding with phosphate groups is also possible. The present review article illustrates a recent account of varied aspects of these two alkaloids and their roles in biological systems.

## Toxicological effects of berberine

On the basis of the amount of berberine present in any compound, rout of administration and type of organism LD50 value varies. Some data accumulated by Kulkarni et al. (1972), which gives following information. The LD50 value of powdered root *Berberis vulgaris* which is known as barberry is 2,600 mg/kg in mice on oral administration (Table 1). On orally administration of root extract fraction of *B. vulgaris*, the LD50 values are 1,280 and 520 mg/kg in rat and mice, respectively (Table 1). In mice the LD50 value of pure berberine on intraperitoneal (IP) and orally administration are 23 and 329 mg/kg, respectively (Table 1). Berberine sulfate isolated from *Berberis aristata* on intraperitoneal administration in rats have LD50 value equal to 205 mg/kg. However, administration of 50 mg/kg of Berberine sulfate causes diarrhea in 40 % of rats which directly effects the gastrointestinal track (Kulkarni et al., 1972).

**Table 1:** LD_50_ of berberine and sanguinarine (of extract and pure compound) as determined in different animals through different routes of exposure (S.No.1-6 Kulkarni et al., 1972 and S.No.7-9 Becci et al., 1987).

**S.No**.	**Extract**	**Animal**	**Rout**	**LD_50_ (mg/kg)**
1	Powdered root *Berberis vulgaris*	Mice	Oral	2,600
2	Root extract fraction of *B. vulgaris*	Rat	Oral	1,280
3	Root extract fraction of *B. vulgaris*	Mice	Oral	520
**S.No**.	**Pure Compound**	**Animal**	**Rout**	**LD_50_ (mg/kg)**
4	Pure berberine	Mice	Oral	329
5	Pure berberine	Mice	Intraperitoneal	23
6	Berberine sulfate isolated from *Berberisaristata*	Rat	Intraperitoneal	205
7	Pure sanguinarine	Rat	Oral (acute)	1,658
8	Pure sanguinarine	Rat	Intra-venous (acute)	29
9	Pure sanguinarine	Rabbit	Dermal (acute)	<200

In cats 100 mg/kg (orally) of berberine evokes vomiting in 6–8 h and the same dose for 8–10 days caused death of all animals. In cats 50/100 mg/kg for 10 days oral administration of berberine sulfate caused hemorrhagic inflammatory problems in both small and large intestine. Some mild symptom of low amount of berberine and its compounds poisoning has been seen in dogs. These symptoms are salivation, nausea, diarrhea, emesis, muscular tremor, and sometimes paralysis also appeared in dogs (Lampe, 1992). The sub-acute toxicity of berberine shows gastric ulcers (Kupeli et al., 2002), Freund's complete adjuvant-induced chronic arthritis, liver and kidney enlargement, increase in body weight (up to 30%) (Yesilada and Kupeli, 2002), decreases bilirubin protein binding in adult rats (Ho et al., 2014). Mahmoudi et al. (2016) has been reported some immunotoxic effects of berberine. It is reported that, 10 mg/kg of berberine administration responsible for reduced number of leukocytes, neutrophils, lymphocytes (blood cell count), and spleen weight. Significant decrease generation/differentiation of B- and T-cells and splenic CD19+ B-cells, CD4+ and CD8+ T-cells is also associated with berberine. Totally, 5 mg/kg of berberine is responsible for only influence the proliferation of lymphocytes and delayed-type hypersensitivity response while 10 mg/kg of berberine is responsible for suppressed both cellular and humoral immune functions (Mahmoudi et al., 2016) (Table 2).

**Table 2:** Dose dependent effects of berberine and sanguinarine.

**S.No**.	**Doses**	**Effects**	**References**
1	50 mg/kg of Berberine sulfate	Affects the gastrointestinal track by inducing diarrhea in rats.	Kulkarni et al., 1972
2	50/100 mg/kg of berberine sulfate for	Causes hemorrhagic inflammatory problems in both small and large intestine after 10 days of exposure in cats.	Lampe, 1992
3	100 mg/kg of berberine	Evokes vomiting (6–8 h) and caused death in cats (8–10 days).	Lampe, 1992
4	10 mg/kg of berberine	Reduces blood cell count (leukocytes, neutrophils, lymphocytes), spleen weight, generation/differentiation of B- and T-cells and splenic CD19+ B-cells, CD4^+^ and CD8^+^T-cells (cellular and humoral immune functions).	Mahmoudi et al., 2016
5	5 mg/kg of berberine	Influence the proliferation of lymphocytes and delayed-type hypersensitivity response.	Mahmoudi et al., 2016
6	50, 100 and 150 mg/kg of berberine	Induces liver tissue damages.	Zhou et al., 2008
7	10 mg/kg of sanguinarine	Increase the activity of SGPT and SGOT as well as it was responsible for the hepatotoxicity and drastic loss in microsomal cytochrome P-450 and benzphetamine N-demethylase activity.	Dalvi, 1985
8	IC_50_ value of 0.9 μM of sanguinarine	Decreases the cell viability in human gingival fibroblasts and triggers mouse embryonic stem cell (ESC) apoptosis in a dose-dependent manner.	Malikova et al., 2006a
9	0.5–2 μM of sanguinarine	Induces apoptosis and exert negative effect on the mouse embryonic development.	Vrba et al., 2009

Sub-chronic toxicity of berberine has reported to damages lung and liver by increasing alanine aminotransferase (ALT) and aspartate aminotransferase (AST), significantly (Ning et al., 2015). In another study on mosquito larvae of *Aedes atropatpus*, effects of berberine showed chronic toxicity and significantly increased cumulative mortality (Philogene et al., 1984). Another study has revealed that in diabetic rats after 16 weeks of berberine administration at concentrations >50, 100, and 150 mg/kg induces liver tissue damages but these symptoms do not appear in healthy rats (Zhou et al., 2008). Berberine in ApoE-/- mice evokes atherosclerosis after IP treatment for 15 weeks with 5 mg/kg/day (Li et al., 2009). Further, exposure to berberine results into uterine contraction and also may lead to teratogenic effects (the substances responsible for inducing developmental toxicity in an organism from the time of conception till birth) (Table 2).

Experimental studies validated by docking studies has been reported the mode of action (through hydrophobic interactions) and inhibitory effect of berberine against main neurological enzymes namely acetylcholinesterase (AChE), butyryl cholinesterase (BChE), and monoamine oxidase (MAO) (Ji and Shen, 2012). An LD50 of berberine that can inhibit AChE, BChE, MAO-A, and MAO-B are 0.44, 3.44, 126, and 98.2 μM, respectively (Ji and Shen, 2012). It was reported that treatment of 10 and 30 μM of berberine exposure to PC12 cells increased cyto-toxicity that was indicated by increase in apoptotic cell death. The *in vitro* (5 and 30 mg/kg, i.p. for 21days) and *in vivo* (10 and 30 μM up to 48 h) studies with berberine against 6-hydroxydopamine (6-OHDA) induced neuro-toxicity in rats and PC-12 cells, respectively, have demonstrated inhibition of dopamine biosynthesis, accompanied by reduced levels of norepinephrine (NE) and dopamine (DA) (Kwon et al., 2010).

## Toxicological effects of sanguinarine

The short-term toxicity of sanguinarine, a benzophenanthridine alkaloid, and two other alkaloids of *S. canadensis* L. extracts have been reported. The acute oral LD_50_ in rats were reported to be about 1,658 mg/kg of sanguinarine. When given through intra-venous route, the acute LD_50_ in rats was observed to be 29 mg/kg of sanguinarine (Table 1). However, the acute dermal LD_50_ of sanguinarine in rabbits was found to be greater than 200 mg/kg (Becci et al., 1987) (Table 1). Occurrences of epidemic dropsy in the tropics have been examined for its hepatotoxic potential in rats which was due to administration of the alkaloid sanguinarine. In some studies, it was found that a single IP dose of about 10 mg/kg of sanguinarine was responsible for increase in the activity of serum glutamic pyruvic transaminase (SGPT) and serum glutamic-oxaloacetic transaminase (SGOT) substantially as well as it was responsible for the drastic loss in microsomal cytochrome P-450 and benzphetamine N-demethylase activity. Furthermore, in the same study, significant decrease in body and liver weights and slightly enlargement in livers with fibrinous material and peritoneal edema were recorded in the treated rats. Progressive degeneration of cells and necrosis has been examined by microscopy in the liver tissue. Further, all these changes substantiating that sanguinarine is a potential hepatotoxic alkaloid (Dalvi, 1985). Moreover, depending on the dosage sanguinarine and its derivative dihydrosanguinarine were found to induce HL60 cell death through apoptotic or necrotic processes (Vrba et al., 2009). In addition, sanguinarine exhibits antiproliferative and pro-apoptotic properties on normal and cancer cells (Slunská et al., 2010).

Some workers have reported the effect of sanguinarine on cell viability. According to this study, IC_50_ value of 0.9 μM of sanguinarine decreased the cell viability in human gingival fibroblasts (Malikova et al., 2006a) determined by using the [3-(4,5-dimethylthiazol-2-yl)-2,5-diphenyltetrazolium bromide] MTT assay after 4 h of exposure (Vrba et al., 2009) (Table 2). Another study revealed that with an IC_50_ value of 0.95 μM, sanguinarine triggered mouse embryonic stem cell (ESC) apoptosis in a dose-dependent manner. The IC_50_ value of sanguinarine was determined by using the MTT assay after 24 h exposure to the material. On the basis of above study, the cytotoxic effects of 0.5–2 μM sanguinarine on pre- and post-implantation embryonic development was determined. Sanguinarine in dose dependent manner caused apoptosis in mouse blastocysts as determined by terminal deoxynucleotidyl transferase dUTP nick end labeling (TUNEL) staining. These workers have also discovered primary occurrence of sanguinarine mediated cellular loss and apoptosis in the inner cell mass (ICM) by dual differential staining. They have demonstrated in their *in vivo* and *in vitro* studies that sanguinarine at 0.5–2 μM may induce apoptosis and exert negative effect on the mouse embryonic development (Chan, 2011). The workers have concluded that sanguinarine even at physiological doses can adversely influence both the pre-implantation and post-implantation embryonic development in rats (Vrba et al., 2009) (Table 2).

Cytotoxicity or antiproliferative properties of sanguinarine in normal or cancer cell lines, such as rat hepatocytes (Choy et al., 2008), human gingival fibroblasts (Malikova et al., 2006b), human osteosarcoma cells (Park et al., 2010), and human promyelocytic leukemia HL-60 cells (Vrba et al., 2009) have been reported by several investigators. A number of alkaloids isolated and evaluated from *A. mexicana* for cytotoxic activity have been evaluated. The *N*-demethyloxysanguinarine was reported to cause nasopharyngeal carcinoma (HONE-1) to human and human gastric cancer (NUGC) cell lines (Chang et al., 2003). In another study, Uddin et al. (2011) revealed the cytotoxic activity against healthy mouse fibroblasts (NIH3T3) and three human cancer-cell lines (AGS, HT-29, and MDA-MB-435S) of methanolic extract of *A. mexicana* leaves by using the MTT assay. One of the cancer cell lines such as MDAMB-435S exhibited more cytotoxic effects of extracts from *A. mexicana* leaves (IC50 1.82 mg/ml) (Brahmachari et al., 2013).

## Conclusion

Berberine and sanguinarine are traditionally used alkaloids with multispectrum pharmacodynamic properties. On the basis of extensive literature survey, berberine, and sanguinarine have been reported to cause toxicity in different living system. In molecular structure of berberine and sanguinarine, both contains a positive moiety which interacts with a number of nucleophilic and anionic moieties of many biomolecules that distort their structure and further resulted in to altered function of biomolecules. Instead of antitumor activity of berberine, it has potential to treat diabetes mellitus. They have been implicated in the occurrence of dropsy. The toxicity of pure compound is greater than the toxicity of plant extract or plant extract fractions. The sub-acute concentrations of berberine lead to altered liver function, gastric troubles, hepato and hematotoxicity, hemorrhagic inflammatory consequences, damage to immune cells and induced apoptosis. The *in vivo* and *in vitro* studies have reported that sanguinarine may induce apoptosis and adversely influence the embryonic development (both in the pre-implantation and post-implantation conditions) of mouse. Sanguinarine toxicity is also reflected in terms of increased SGPT and SGOT activities and reduced microsomal cytochrome P-450 and benzphetamine N-demethylase activities. On the other hand, the cytotoxic properties of both of these alkaloids reveal the use of these alkaloids in treatment of cancer. Berberine treatment may improve insulin resistance, promote insulin secretion, inhibit gluconeogenesis in liver, stimulate glycolysis in peripheral tissue cells, modulate gut microbiota, reduce intestinal absorption of glucose, and perturb lipid metabolism. However, more work is required to assess their anticancer potential under different environmental and clinical conditions to ascertain this possibility.

## Author contributions

NS: Wrote the review article prepared and assembled the figure and table; BS: Critically organized and revised the manuscript by incorporating significant reports.

### Conflict of interest statement

The authors declare that the research was conducted in the absence of any commercial or financial relationships that could be construed as a potential conflict of interest.
